# Postconditioning with Irisin Attenuates Lung Ischemia/Reperfusion Injury by Suppressing Ferroptosis via Induction of the Nrf2/HO-1 Signal Axis

**DOI:** 10.1155/2022/9911167

**Published:** 2022-03-02

**Authors:** Yun Wang, Zhe Dong, Zongze Zhang, Yanlin Wang, Kun Yang, Xinyi Li

**Affiliations:** ^1^Department of Anesthesiology, Zhongnan Hospital of Wuhan University, Wuhan, Hubei, China; ^2^Department of Cardiovascular Surgery, Zhongnan Hospital of Wuhan University, Wuhan, Hubei, China; ^3^Zhongnan Hospital of Wuhan University, Wuhan, Hubei, China

## Abstract

Iron-dependent lipid peroxidation causes ferroptosis. This study was aimed at verifying that irisin postconditioning can inhibit ferroptosis and minimize lung ischemia/reperfusion (I/R) damage via activating the Nrf2/HO-1 signal axis. We constructed a murine model of I/R lung damage. At the onset of reperfusion, irisin, ferroptosis inhibitor ferrostatin-1, and ferroptosis inducer Fe-citrate were all administered. We discovered that irisin could reduce lung I/R injury, consistent with ferrostatin-1's action. Furthermore, irisin suppressed ferroptosis in lung I/R damage, as evidenced by lower ROS, MDA, and Fe^2+^, as well as alterations in critical protein expression (GPX4 and ACSL4). However, Fe-citrate abolished the protective effects of irisin. Transcriptome research found that irisin increased the mRNA levels of Nrf2 and HO-1. Thus, we used siRNA to investigate the role of the Nrf2/HO-1 axis in irisin-mediated protection against hypoxia/reoxygenation (H/R) damage in MLE-12 cells. Irisin consistently reduced ferroptosis and improved mitochondrial dysfunction caused by H/R. Irisin's cytoprotective function was eliminated when Nrf2 was silenced. As a result, irisin postconditioning may protect against lung I/R damage by suppressing ferroptosis via the Nrf2/HO-1 signaling axis.

## 1. Introduction

Lung ischemia/reperfusion injury (LIRI), which is linked to poor patient outcomes, occurs in many medical situations, including lung transplantation, pulmonary embolism, and cardiopulmonary bypass [1–3]. Ischemia/reperfusion (I/R) events, or hypoxia/reoxygenation (H/R) in vitro, can produce a large number of oxygen species, which not only recruit proinflammatory cytokines but also damage epithelial and endothelial barriers, potentially leading to pulmonary edema and abnormal gas exchange [4]. Emerging evidence confirms that I/R-induced tissue/cell injury involves regulated cell death (RCD), such as autophagy, necroptosis, and ferroptosis [5–7].

Ferroptosis, a newly discovered type of RCD, is caused by iron-dependent lipid peroxidation [8]. Unlike the traditional kind of nonapoptotic cell death, the primary morphological traits of ferroptosis are mitochondrial shrinkage and increased mitochondrial membrane density [8]. Biochemically, the mechanism underlying ferroptosis is mainly related to glutathione (GSH) depletion, inactivation of glutathione peroxidase 4 (GPX4), iron overload, and lipid peroxidation [9]. Many studies have found that ferroptosis is implicated in various diseases, including I/R-related insults [10–13]. Recent research has found a link between ferroptosis and lung damage. According to Qiu et al., Nrf2 attenuated seawater drowning-induced lung damage by suppressing ferroptosis [14]. According to Dong et al., ferroptosis contributed to lung damage due to intestinal I/R, and Nrf2 inhibited ferroptosis by upregulating SLC7A11 and HO-1 [15]. According to Li et al., an inhibitor of p53 protein prevented ferroptosis and reduced the acute lung injury caused by intestinal I/R through the Nrf2 pathway [16]. These findings suggest that ferroptosis modulated by Nrf2 could be a novel pathophysiological mechanism of LIRI.

Irisin is a hormone-like molecule secreted primarily by skeletal muscles during exercise [17]. Irisin promotes the browning of white adipose tissues and helps to modulate glucose and lipid homeostasis [17–19]. It has been regarded as a novel therapeutic target for metabolic disorders [20–22]. Recently, irisin's antioxidative, anti-inflammatory, and antiferroptosis activities have received much attention. Mazur-Bialy et al. discovered that irisin diminished free radical generation by macrophages via activating the Nrf2 pathway [23]. Tsai et al. proposed that the p62/Nrf2 pathway was responsible for irisin's browning effect [24]. Wei et al. found that irisin might suppress ferroptosis and restore mitochondrial function in sepsis [25]. Moreover, irisin plays a vital role in ischemic preconditioning- (RIPC-) mediated lung protection, and exogenous irisin treatment protects against LIRI by improving mitochondrial function [26]. However, the mechanism by which irisin protects LIRI remains unknown. Ferroptosis is tightly linked to mitochondrial function. However, little is known about the link between irisin and ferroptosis in LIRI. As a result, this study was aimed at exploring the beneficial effect of irisin postconditioning in an experimental LIRI model in vivo and in vitro and the mechanisms associated with ferroptosis related to the Nrf2/HO-1 signal axis.

## 2. Materials and Methods

### 2.1. Animals

C57BL/6 male mice (weighing 20–25 g) were purchased from the Wuhan University's Animal Center (Wuhan, China). All mice were fed standard food and water and kept in a temperature and humidity-controlled environments with a 12-hour light-dark cycle.

### 2.2. Murine LIRI Model

In vivo, the LIRI model was established as described earlier [27]. All mice were anesthetized with pentobarbital administered intraperitoneally (50 mg/kg, Sigma-Aldrich, MO, USA). After endotracheal intubation, the mice were ventilated using a rodent ventilator (MiniVent, Harvard Apparatus, USA), with the title volume set to 7 ml/kg, the respiratory rate set to 120 times/min, and the inspiratory/expiratory ratio set to 1: 2. A noninvasive clamp was used to interrupt the left pulmonary hilum, causing lung ischemia. The clamp was released after 60 minutes of ischemia, and the left lung was reperfused for 120 minutes. Animals were euthanized via cervical dislocation at the end of the experiment. Following that, lung specimens and bronchoalveolar lavage fluid were harvested for analysis. All procedures except lung ischemia were performed on mice in the sham group.

### 2.3. Treatment Protocols

All mice were randomly allocated to six groups: sham group, I/R group, Fer-1 (ferrostatin-1, a ferroptosis inhibitor) group, Fe (Fe-citrate, a ferroptosis inducer) group, Ir (Irisin) group, and Fe+Ir group.

Firstly, to detect the effect of ferroptosis in LIRI, mice in the Fer-1 group and Fe group were given intravenous injections of Fer-1 (1.5 mg/kg, Sigma-Aldrich, MO, USA) and Fe (15 mg/kg, Sigma-Aldrich, MO, USA) at the onset of reperfusion, respectively. The mice in the Ir group were then given an intravenous injection of irisin (250 *u*g/kg, 067-29A, Phoenix Pharmaceuticals, Inc., Burlingame) at the onset of reperfusion to test the efficacy of irisin on LIRI. Finally, the mice in the Fe+Ir group received an intravenous injection of Fe and irisin at the onset of reperfusion to assess the role of irisin in ferroptosis. Fer-1 and Fe were dissolved initially in DMSO and later in normal saline. As previously described, the doses of irisin, Fer-1, and Fe were given [28, 29].

### 2.4. Histopathological Analysis

The samples of the left lung were dissected and fixed with paraformaldehyde. Following that, lung samples were paraffin-embedded and stained with hematoxylin and eosin after being cut into 4-*μ*m slices. Last, the severity of lung damage was assessed independently by two technicians who were blinded to group treatment protocols according to the criteria as recently published [29]. The scoring system was comprised of four components: lung interstitial edema, neutrophil infiltration, alveolar edema, and alveolar congestion. Scores from 0 to 4 were given out as follows: 0 represents normal lung; 1 represents mild damage, insults involving less than 25% of the lung; 2 represents moderate damage, insults that included 25%–50% of the lung; 3 represents severe damage, insults that included 25%–50% of the lung; and 4 represents extreme damage, insults that included more than 75% of the lung. The lung injury score was computed by summing the individual scores for each criterion.

### 2.5. Lung Wet/Dry Mass Ratio

The left lung tissues were removed and weighed instantly after 120 minutes of reperfusion to determine the wet weight. The lung tissues were then set in a 60°C oven for three days and weighed again until a constant mass was reached. To assess the severity of lung edema, we calculated the wet/dry mass ratio.

### 2.6. Detection of Protein Concentration in Bronchoalveolar Lavage Fluid (BALF)

The left lung BALF was harvested at the end of the experiment. According to the manufacturer's protocols, the protein content in BALF was measured by the commercial bicinchoninic acid (BCA) protein assay kit (Beyotime, Shanghai, China).

### 2.7. Transmission Electron Microscopy (TEM)

The samples from the left lung were removed and fixed in glutaraldehyde. Subsequently, the fixed tissues were dehydrated with a graded ethanol series and sliced into ultrathin slices. The slices were then stained with uranyl acetate and lead citrate. Finally, the slices were examined with an HT-7500 transmission electron microscope (Hitachi Co., Tokyo, Japan).

### 2.8. Detection of ROS Levels

The levels of ROS in lung tissues were assessed using the dihydroethidium (DHE) fluorescent probe (D7008, Sigma-Aldrich, MO, USA) following the previously described protocols [30]. In brief, frozen sections were stained with 50 *u*M of DHE for 1 hour at room temperature in the dark and then with DAPI (1 mg/ml) for 10 minutes. The fluorescence intensity was measured using a fluorescent microscope (TE-2000, Nikon Co., Tokyo, Japan) at 525 nm excitation (Ex) wavelength and 610 nm emission (Em) wavelength after washing.

ROS levels in MLE-12 cells were tested by the 2′, 7′-dichlorofluorescein diacetate (DCFH-DA) fluorescent probe (D6883, Sigma-Aldrich, MO, USA) following the manufacturer's protocols. After treating MLE-12 cells with 10 *u*M DCFH-DA for 1 hour in the dark and then treated with DAPI (1 ug/ml) for 10 minutes, the cell pictures were detected using a fluorescent microscope (TE-2000, Nikon, Co., Tokyo, Japan) at (Ex/Em) 485 nm and 530 nm.

### 2.9. Determination of Related Indicators

The levels of malondialdehyde (MDA), glutathione (GSH), and ferrous ions in tissue lysates and MLE-12 cells were determined by the commercial MDA assay kit (Beyotime, Shanghai, China), GSH assay kit (Beyotime, Shanghai, China), and iron assay kit (MAK025, Sigma-Aldrich, MO, USA) according to the respective manufacturer's protocols.

### 2.10. Western Blot

We performed western blot as previously recorded [27]. Proteins were extracted from lung tissues or cells and were then lysed in a RIPA buffer containing protease and phosphatase inhibitors. The BCA kit quantified the protein content (Beyotime, Shanghai, China). 50 *μ*g of protein samples from each group (Nrf2 in the nuclear fraction, HO-1 in the cytosolic fraction, and others from both fractions) was electrophoresed in a 10% SDS-PAGE gel and transferred to a PVDF membrane. The blots were incubated with the primary antibodies against GPX4 (1: 1000, ab125066, Abcam), ACSL4 (1: 1000, ab155282, Abcam), Nrf2 (1: 1000, ab62352, Abcam), HO-1 (1: 1000, ab13248, Abcam), histone H2A (1: 1000, ab177308, Abcam), or GAPDH (1: 1000, ab181602, Abcam) overnight at 4°C after blocking with 5% nonfat dry milk. After that, the blots were washed in TBST and incubated for 2 hours at room temperature with a secondary antibody. The bands were identified by an ECL system and an ECL kit (Beyotime, Shanghai, China), and band intensities were checked using the ImageJ software.

### 2.11. Cell Culture

MLE-12 cells (mouse lung epithelial cell line, obtained from ATCC) were cultured in DMEM (Gibco, Grand Island, USA) with 10% fetal bovine serum (DMEM, Gibco, Grand Island, USA) and kept in a 37°C incubator with 5% CO_2_.

### 2.12. H/R Model in MLE-12 Cells

The H/R model was modified from a previous study [29]. In brief, after being starved in a serum-free DMEM for 12 hours, the cells were incubated in an anaerobic incubator (Thermo Fisher Scientific, MA, USA) containing 95% N2 for 8 hours to induce hypoxia. The dishes were replaced with a standard medium for reoxygenation and transferred to a normoxic incubator for 12 hours.

Firstly, to explore the effect of ferroptosis, MLE-12 cells were incubated with Fer-1 (0.1 *u*M) or Fe (3.3 mM) at the beginning of reoxygenation. Secondly, to detect the role of irisin, the cells were incubated with irisin (8 nM) before reoxygenation. Thirdly, to confirm the function of irisin on ferroptosis, MLE-12 cells were coincubated with irisin and Fe before reoxygenation. Lastly, to evaluate the role of Nrf2, either control siRNA (si-con) or Nrf2 siRNA (si-Nrf2; GenePharma Co., Ltd., Shanghai, China) was transfected into MLE-12 cells for 48 hours before H/R using the Lipofectamine 2000 TM reagent (Invitrogen, USA).

### 2.13. Assay for Cell Viability

As previously illustrated, cell viability was tested using the CCK-8 assay kit (Beyotime, Shanghai, China) [14]. Briefly, MLE-12 cells were seeded into 96-well plates at a density of 6 × 10^3^ cells per well and incubated for 24 hours before being subjected to various treatments. Following that, 10 *u*l of CCK8 solution was added to each well and cultivated for 3 hours. The absorbance at 450 nm was measured by a microplate reader (PerkinElmer, USA).

### 2.14. Assay for Lipid ROS

AS previously illustrated, Lipid ROS was detected using the BODIPY™ 581/591 C11 (D3861, Invitrogen) fluorescent probe [31]. First, the MLE-12 cells were incubated with 2 *u*M of BODIPY 581/591 C11 for 1 hour at 37°C. Subsequently, the cells were washed with PBS, trypsin digested, and suspended in PBS. Finally, cell images were analyzed by flow cytometry (BD Accuri C6 plus, BD Biosciences, USA).

### 2.15. Mitochondrial Membrane Potential (MMP) Assay

The MMP of MLE-12 cells was measured using the JC-1 fluorescent probe (CAS 3520-43-2, Abcam). The cells were incubated with five *μ*M JC-1 for 30 minutes in the dark at 37°C. After washing with PBS, cell images were detected using a fluorescent microscope (TE-2000, Nikon Co., Tokyo, Japan).

### 2.16. Mitochondrial Ferrous Iron (Fe^2+^) Determination

The mitochondrial Fe^2+^ of MLE-12 cells was examined using the Mito-Tracker Green fluorescent probe (Beyotime, Shanghai, China). The medium was removed. The cells were then treated with 5 *μ*M Mito-FerroGreen working liquid in the dark for 30 minutes. The working fluid was then removed, and a fresh DMEM medium was added to the cells. Finally, the cell images were examined using a confocal microscope (TCS-SP2, Leica, Germany) at Ex and Em wavelengths of 488 nm and 510–550 nm, respectively.

### 2.17. RNA Sequence Analysis

RNA was isolated from the lung tissue of different groups. The Implant NanoPhotometer® spectrophotometer detected the purity of RNA. According to the manufacturer's instructions, sequencing libraries were developed using the RNA Library Prep Kit for Illumina® (NEBNext® Ultra, NEB, USA). The AMPure XP system purified the PCR products, and the library quality was checked using the Agilent Bioanalyzer 2100 system. The HiSeq 4000 PE Cluster Kit (Illumina, USA) was performed to cluster the index-coded samples following the manufacturer's protocols. Finally, the library preparations were sequenced on a HiSeq 4000 platform for 150 cycles. All library construction and sequencing steps were performed at Shanghai Life Gene Technology Co., Ltd. The DEGseq R package was used for differential expression analysis of two groups. Genes with a log2 fold change of >0.58 and *P* < 0.05 were considered differentially expressed genes (DEGs). GO enrichment analysis of DEGs was performed using the DAVID database.

### 2.18. Statistical Analysis

All values are shown as the mean ± standard deviation. One-way ANOVA analyzed differences among multiple groups. *P* < 0.05 was considered statistically significant.

## 3. Results

### 3.1. The Effects of Various Irisin Dose Levels on LIRI

The results of HE staining revealed that the lung tissue of the sham mice had typically organized architecture with no edema, lesions, or neutrophils. I/R, on the other hand, caused significant pathological injury, including interstitial edema, alveolar hemorrhage, and massive inflammatory cell infiltration (Figure S1A). A degree of relief was seen in the low-dose irisin group, and a greater degree of relief was seen in the high-dose irisin group (Figures S1A and S1B). Hypoxemia is yet another symptom of lung injury. In mice subjected to lung I/R injury, arterial blood gas analyses revealed that PaO_2_ was decreased and PaCO_2_ was increased, but irisin administration improved these hallmarks of lung function, with the high-dose providing the perfect function (Figures S1C and D). The results showed that irisin could improve LIRI, and the effect was most significant when the irisin dose was 250 *u*g/kg. As a result, the subsequent irisin experiments were carried out at a dose of 250 *u*g/kg.

### 3.2. Irisin Attenuates Ferroptosis during LIRI

**Figure 1:**
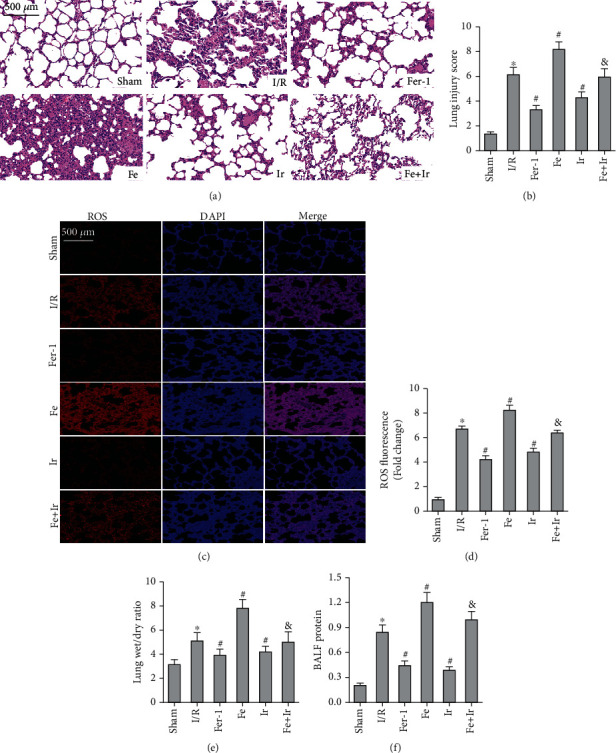
Irisin attenuates I/R-induced lung injury. Mice were given a sham operation or lung I/R (60 minutes of ischemia followed by 120 minutes of reperfusion) with or without administration of Ir, Fer-1, and Fe. (a) Representative images (200x) of HE staining in the lung sections (scale bar = 500 *μ*m). (b) The degree of lung injury. (c and d) The ROS production in lung tissues was measured by the DHE fluorescence probe (scale bar = 500 *μ*m). (e) Lung wet/dry mass ratio. (f) The protein level of BALF. ^∗^*P*< 0.05 vs. sham group, ^#^*P* < 0.05 vs. I/R group, and ^&^*P* < 0.05 vs. Fe group. The values are shown as mean ± SD (*n* = 6).

To confirm the role of ferroptosis in LIRI, we examined the impact of Fer-1 administration. As shown in Figures 1(a) and 1(b), I/R produced significant pathological changes. However, Fer-1 treatment alleviated pathological changes caused by I/R. Another critical indicator of lung injury was noncardiogenic pulmonary edema. As illustrated in Figures 1(e) and 1(f), the administration of Fer-1 significantly mitigated I/R-stimulated lung edema as evidenced by lung W/D mass ratio and protein leakage in BALF. These results confirmed the presence of ferroptosis in LIRI.

Irisin postconditioning reduced LIRI in the same way that Fer-1 treatment did. However, iron supplementation with Fe-citrate exacerbated LIRI, resulting in severe pathological lesions and pulmonary edema, which were attenuated by irisin (Figures 1(a) and 1(b), Figures 1(e) and 1(f)).

Inflammation has a vital role in the progression of LIRI [32]. Moreover, the inflammatory response caused by I/R can result in microvascular leakage. Hence, we collected BALF and determined its proinflammatory factors. The use of Fer-1 and irisin treatment essentially diminished the increase of TNF-*α*, IL-1*β*, and IL-6 content in BALF caused by I/R (Figures S2).

**Figure 2:**
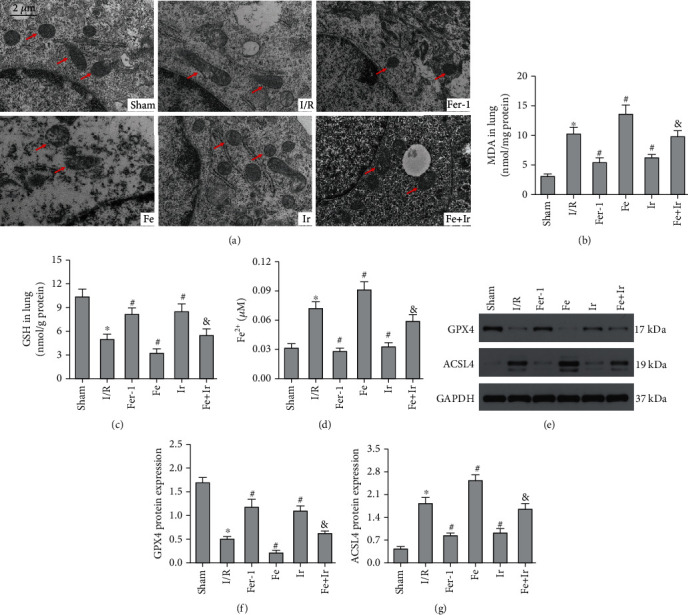
Irisin prevents ferroptosis following lung I/R damage. (a) Representative transmission electron microscopy images (scale bar = 2 *μ*m). The red arrow represents representative mitochondria in the lung of a mouse (*n* = 3 mice/group). MDA (b), GSH (c), and Fe^2+^ (d) contents in mouse lung homogenates. (*n* = 6). (e) Western blot examination of the proteins GPX4 and ACSL4 in the lung tissue. (*n* = 6). Quantitative results are shown in (f) and (g). ^∗^*P* < 0.05 vs. sham group, ^#^*P* < 0.05 vs. I/R group, and ^&^*P* < 0.05 vs. Fe group. The values are shown as mean ± SD.

Ferroptosis is a newly discovered type of RCD characterized by iron-dependent accumulation of lipid peroxidation [8]. Furthermore, ferroptosis appears to play a significant role in inflammation [33]. To demonstrate that irisin's protection against LIRI is related to ferroptosis, we measured the tissue ROS generation and lipid ROS levels (MDA and GSH). I/R markedly increased ROS and MDA levels while decreasing GSH activity, whereas Fer-1 and irisin postconditioning inhibited the oxidative damage caused by I/R (Figures 1(c) and 1(d), Figures 2(b) and 2(c)). Iron content and essential ferroptosis-related protein (GPX4 and ACSL4) expression were then determined. The data showed that iron concentration and the expression of the proferroptosis protein ACSL4 increased during I/R, while the expression of the antiferroptosis protein GPX4 declined. Postconditioning with Fer-1 and irisin, on the other hand, reduced these changes (Figures 2(d)–2(g)). The morphological characteristics of ferroptosis were evaluated using TEM. Accordingly, I/R induced significant morphological changes, including smaller mitochondria and cristae reduction, whereas Fer-1 and irisin alone ameliorated I/R-induced mitochondrial morphologic changes (Figure 2(a)).

Overall, these data suggested that ferroptosis contributed to LIRI and that irisin postconditioning mitigated LIRI by inhibiting ferroptosis.

### 3.3. Irisin Inhibits H/R-induced Cell Damage and Ferroptosis in MLE-12 cells

**Figure 3:**
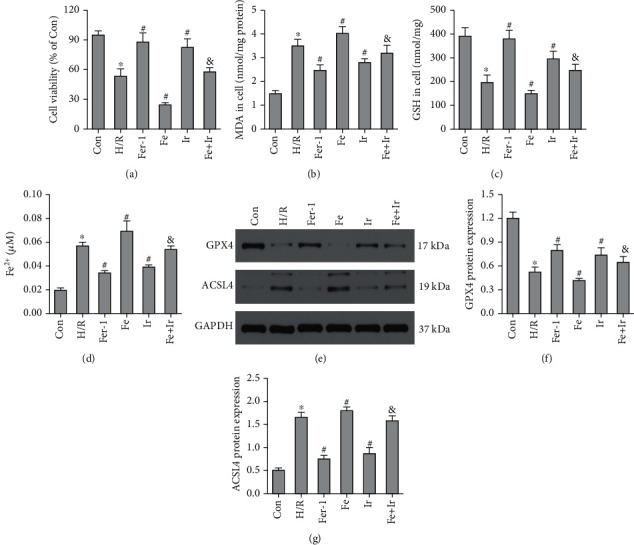
Irisin inhibits H/R-induced ferroptosis in MLE-12 cells. (a) The CCK-8 test kit was used to evaluate cell viability. MDA (b), GSH (c), and Fe^2+^ (d) contents in MLE-12 cells were determined using the relevant kits. (e) Western blot examination of the proteins GPX4 and ACSL4 in MLE-12 cells. Quantitative analysis of findings in (f) and (g). ^∗^*P* < 0.05 vs. Con group, ^#^*P* < 0.05 vs. H/R group, and ^&^*P* < 0.05 vs. Fe group. The values are shown as mean ± SD (*n* = 6).

Based on the in vivo results, we investigated whether irisin postconditioning could prevent H/R-induced cell damage and ferroptosis in MLE-12 cells. The CCK-8 test kit was used to evaluate cell viability. The results indicated that H/R could decrease cell viability (Figure S3A). H/R consistently increased the release of LDH and MDA in MLE-12 cells. Irisin inhibited H/R-induced cell damage dose dependently (Figures S3B and C). We chose 8 nM irisin or the subsequent studies. As seen in Figure 3(a), irisin and Fer-1 alone blocked the loss of cell death caused by H/R. Irisin postconditioning, like Fer-1 alone, inhibited the H/R-induced increase in MDA, Fe^2+^, and ACSL4 expression while also preventing the decrease in GSH activity and GPX4 expression (Figures 3(b)–3(g)). Notably, irisin abrogated the function of Fe-citrate on H/R damage (Figures 3(a)–3(g)). These data indicated that irisin suppressed H/R-induced ferroptosis in vitro.

### 3.4. Irisin Upregulates the Nrf2/HO-1 Signaling Pathway

**Figure 4:**
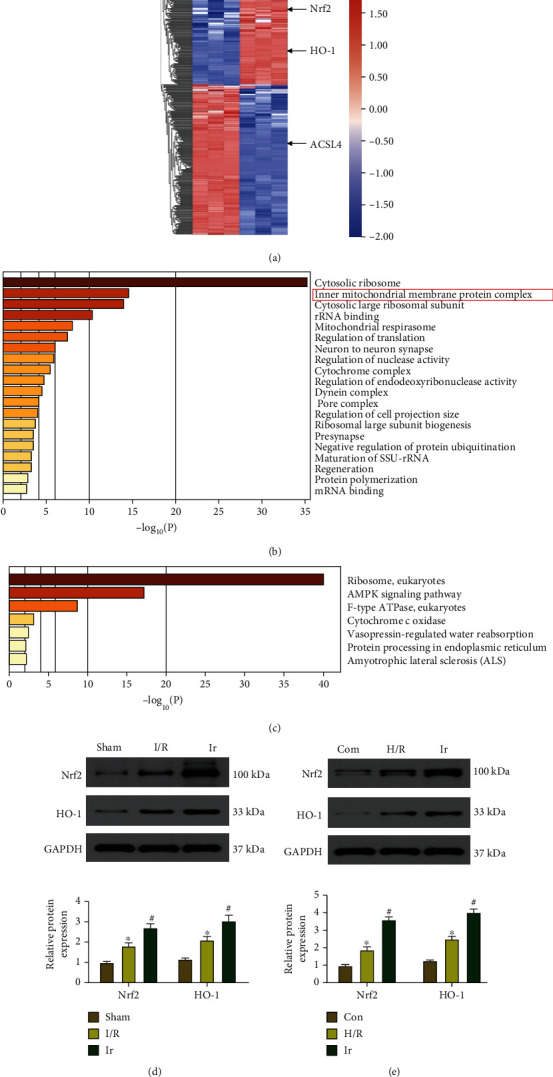
Irisin activates the Nrf2/HO-1 pathway. (a) A heatmap of DEGs in I/R lung tissues treated with irisin and not treated with irisin. (b) Gene Ontology enrichment was based on differentially expressed proteins with a *P*-value smaller than 0.05 using Metascape: upregulated signaling pathway. (c) Downregulated signaling pathway in Gene Ontology enrichment. (d) Protein quantification of Nrf2 and HO-1 in lung tissues using Western blots. (e) Protein quantification of Nrf2 and HO-1 in MLE-12 cells using Western blots. ^∗^*P* < 0.05 vs. Con group, ^#^*P* < 0.05 vs. I/R group. The values are shown as mean ± SD (*n* = 6).

RNA sequencing (RNA-seq) was used to quantify the irisin treatment gene expression profiles. Transcriptome analysis revealed that irisin markedly upregulated the mRNA levels of Nrf2 and HO-1 in the context of LIRI (Figures 4(a)–4(c)). The protein expression of Nrf2 and HO-1 was analyzed by western blot. As shown in Figure 4(d), I/R stimulation upregulated nuclear Nrf2 and cytosolic HO-1 expression in the lung tissue, while irisin further upregulated nuclear Nrf2 and cytosolic HO-1 expression. The same results were obtained in the H/R model (Figure 4(e)).

### 3.5. Irisin Inhibits I/R-induced Ferroptosis through the Nrf2/HO-1 Pathway

**Figure 5:**
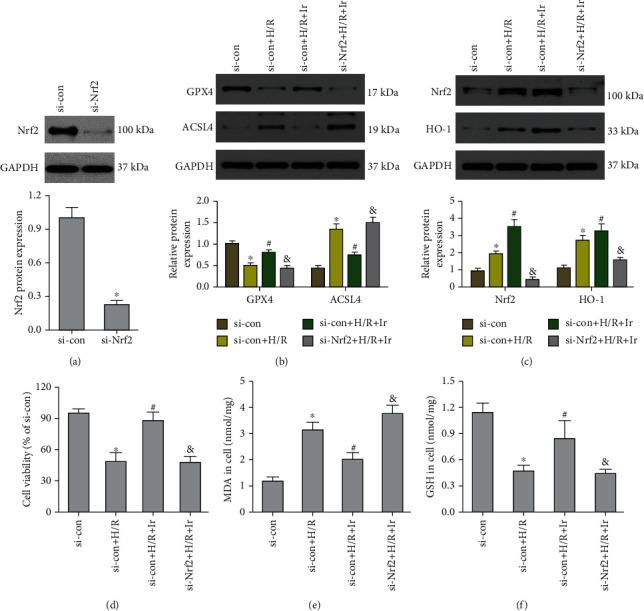
Irisin suppresses ferroptosis produced by H/R via the Nrf2/HO-1 pathway. MLE-12 cells were transfected with Nrf2 siRNA (si-Nrf2) or a negative control before exposure to H/R and irisin. (a) Western blot examination of the Nrf2 protein in cells. (b) Protein quantification of GPX4 and ACSL4 proteins in cells. (c) Protein quantification of Nrf2 and HO-1 proteins in cells. (d) The CCK-8 test kit was used to evaluate cell viability. MDA (e) and GSH (f) levels in cells were analyzed using the relevant kits. ^∗^*P* < 0.05 vs. si-con group, ^#^*P* < 0.05 vs. H/R group, and ^&^*P* < 0.05 vs. si-con+H/R+Ir group. The values are shown as mean ± SD (*n* = 6).

**Figure 6:**
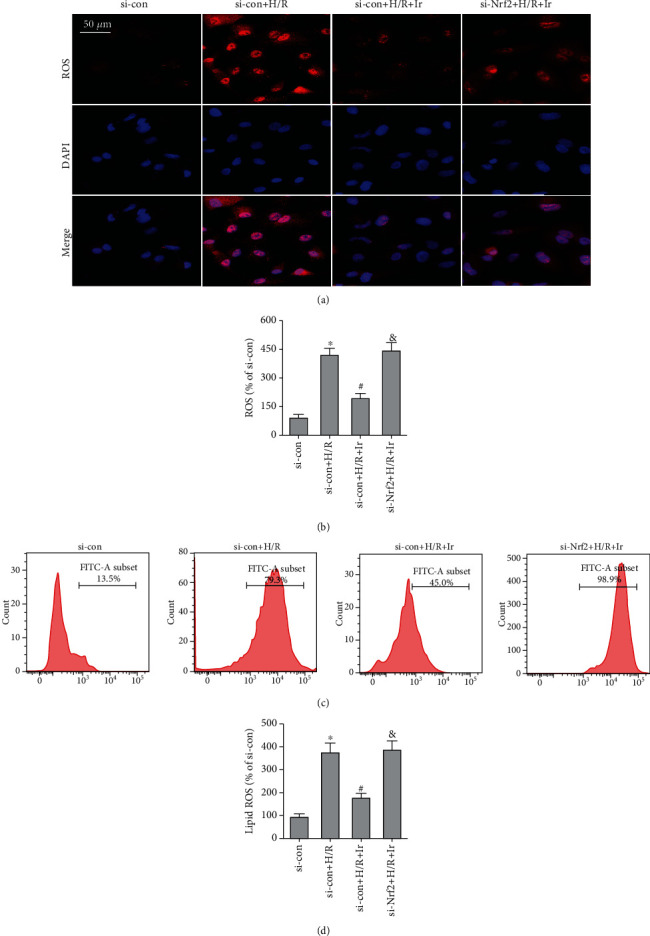
Irisin inhibits H/R-induced ROS production and lipid peroxidation. (a and b) Intracellular ROS was measured using the fluorescent DCFH-DA probes (Bar = 50 *μ*m). (c and d) Lipid ROS was measured using the BODIPY® 581/591 C11 fluorescent probe (*n* = 3). ^∗^*P* < 0.05 vs. si-con group, ^#^*P* < 0.05 vs. H/R group, and ^&^*P* < 0.05 vs. si-con+H/R+Ir group. The values are shown as mean ± SD (*n* = 6).

To further investigate the involvement of the Nrf2/HO-1 axis in irisin-mediated protection against H/R injury, we employed Nrf2-siRNA to knockdown Nrf2 expression. As shown in Figure 5(a), Nrf2 expression was markedly decreased following transfection. Irisin postconditioning prevented the loss of cell viability and oxidative damage caused by H/R, whereas si-Nrf2 transfection eliminated this effect (Figures 5(d)–5(f)). Consistently, si-Nrf2 transfection restored the effect of irisin on the change in ACSL4 and GPX4 expression induced by H/R (Figures 5(b) and 5(c)). The DCFH-DA fluorescent probe evaluated intracellular ROS levels in MLE-12 cells after H/R. The results revealed that irisin lowered ROS levels, whereas si-Nrf2 transfection raised ROS levels (Figures 6(a) and 6(b)). The lipid ROS results also indicated that si-Nrf2 transfection reversed the protective effect of irisin on lessening lipid ROS accumulation stimulated by H/R (Figures 6(c) and 6(d)).

**Figure 7:**
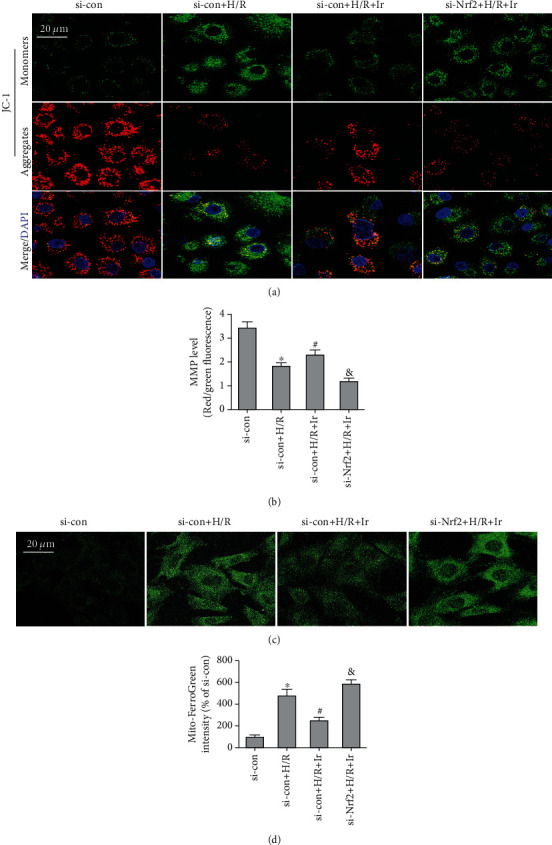
Irisin improves mitochondrial function. (a) Fluorescence photos of MLE-12 cells stained with JC-1 (Scale bars = 20 *μ*m). (*n* = 3). (b) Quantitative results of MMP (c) Fluorescence photos of MLE-12 cells stained with Mito-FerroGreen (Scale bars = 20 *μ*m). (d) Quantitative results of mitochondrial ferrous iron. ^∗^*P* < 0.05 vs. si-con group, ^#^*P* < 0.05 vs. H/R group, and ^&^*P* < 0.05 vs. si-con+H/R+Ir group. The values are shown as mean ± SD (*n* = 6).

Mitochondria are essential for redox homeostasis and ferroptosis [34]. In MMP experiments, irisin improved H/R-induced MMP decrease, whereas si-Nrf2 transfection reversed this (Figures 7(a) and 7(b)). Similarly, the results of Mito-FerroGreen staining indicated that si-Nrf2 transfection reversed the function of irisin on decreasing free iron ions in mitochondria. The above results implied that irisin inhibited H/R-stimulated ferroptosis through the Nrf2/HO-1 pathway (Figures 7(c) and 7(d)).

## 4. Discussion

This study focused on the role of irisin in ferroptosis and its possible therapeutic effects on LIRI. The significant findings of the study are as follows: (1) ferroptosis is involved in LIRI both in vivo and in vitro; (2) irisin postconditioning protects against LIRI by inhibiting ferroptosis; and (3) the protective mechanism of irisin on LIRI is dependent on the Nrf2/HO-1 pathway.

LIRI is a complex inflammatory process that includes epithelial and endothelial damage, the release of inflammatory mediators, and overwhelming innate immune responses [32]. The majority of these responses are driven by the rapid and robust production of reactive oxygen species (ROS), which cause tissue/cell damage and the activation of signaling pathways that promote inflammation and cell death [32]. Irisin is a novel muscle-derived myokine closely associated with metabolic disorders due to its energy expenditure and metabolic properties [17]. Recently, the effect of irisin on ROS has attracted substantial attention. Studies have revealed that irisin can protect human umbilical vein endothelial cells from high-glucose-induced oxidative injury [35]. In addition, irisin has been proved to boost the antioxidant enzyme activities and reduce oxidative damage in patients with gestational diabetes [36]. This study demonstrated that irisin postconditioning could alleviate I/R-induced lung pathological change, reduce lung edema and oxidative damage, and inhibit the release of inflammatory factors in BALF. This result is consistent with earlier research demonstrating that irisin protects against oxidative stress in I/R-injured lung tissue [26]. We further demonstrated the protective mechanism of irisin on ferroptosis induced by I/R.

Ferroptosis, a novel type of RCD, is characterized by iron-dependent accumulation of lipid peroxidation and can be rescued by lipid peroxidation inhibitors (Fer-1) or lipophilic antioxidants [8, 37]. Cells contain two forms of iron: Fe^2+^ and Fe^3+^. Both are essential for appropriate iron homeostasis. Ferroportin and transferrin receptors (TFR) regulate iron homeostasis. TFR-transferrin complexes transport Fe^3+^ into the endosome, which is converted to Fe^2+^ before being released to a labile iron pool (LIP). Fe^2+^ is critical for the development of ferroptosis because it oxidizes lipids in the cellular membranes via the Fenton reaction and catalyzes lipid peroxidation via lipoxygenases (LOXs) [37]. This study found that I/R induced ferroptosis-like morphological changes and increased Fe^2+^ concentration and lipid ROS in lung tissue and MLE-12 cells. However, these effects were suppressed by irisin postconditioning and Fer-1. These findings indicate that ferroptosis is involved in LIRI and that irisin postconditioning decreases I/R-induced ferroptosis through modulating Fe^2+^ and lipid peroxidation levels.

We also evaluated other vital regulators of ferroptosis. Cysteine/glutamate antiporter System Xc- is known for synthesizing GSH [37]. Several redox enzymes utilize GSH to reduce lipid ROS, including GPX4. Inhibition of GPX4 or System Xc- may result in lipid peroxide accumulation, thus leading to ferroptosis. This study showed that irisin increased GSH levels and GPX4 expression in I/R-injured lung tissues and cells. This conclusion supports a prior study that found the GSH/GPX4 axis to be responsible for lipid oxidation-induced acute kidney injury and cell death [38]. The Acyl-CoA synthetase long-chain family (ACSL4) is a crucial lipid metabolism enzyme that regulates the synthesis of polyunsaturated fatty acids (PUFA). It can catalyze the formation of arachidonic acid (AA)-CoA or adrenal (ADA)-CoA, which is required for the formation of phospholipid hydroperoxide (PL-OOH) [37]. ACSL4 expression is substantially lower in ferroptosis-resistant cells than in ferroptosis-sensitive cell lines [39]. This study also discovered that ACSL4 expression was elevated following lung I/R or H/R. However, irisin treatment effectively decreased ACSL4 expression. Based on these investigations, we presumed that the antioxidant activity of irisin on LIRI might relate to the inhibition of ferroptosis. We performed rescue experiments with the ferroptosis inducer Fe-citrate (Fe) to prove this hypothesis. As we expected, Fe aggravated LIRI, resulting in more significant histopathological destruction, which was alleviated by irisin postconditioning, indicating that irisin could counteract the effect of excessive iron. These findings suggest that irisin's protective effect against LIRI is partially obtained by inhibiting ferroptosis.

Mitochondria are the core of redox homeostasis and play a central role in regulating ferroptosis [34]. The primary function of mitochondria is to supply energy to cells via oxidative phosphorylation [29]. Moreover, it is a crucial organelle in amino acid, iron, and lipid metabolism. Mitochondrial dysfunction and metabolic changes induced by diverse intracellular and extracellular stimuli determine the fate of cells. A variety cellular metabolic pathways, including amino acid, iron, and lipid metabolism, can initiate ferroptosis [34]. Accumulating evidence suggests that the association between ferroptosis and mitochondria is mediated by the alteration of MMP and the regulation of mitochondrial permeability transition pore (MPTP) [34]. This study monitored mitochondrial function via the MMP and the mitochondrial Fe^2+^ assays. Our results showed that H/R led to a decrease in MMP and increased mitochondrial Fe^2+^ in MLE-12 cells. However, irisin reversed the decline in MMP and reduced mitochondrial iron levels. These results imply that mitochondria play an essential role in irisin's antiferroptosis.

To further explore the protective mechanism of irisin postconditioning on LIRI, we performed a transcriptome study by RNA-seq. Transcriptome analysis uncovered that irisin markedly elevated the mRNA levels of Nrf2 and HO-1, suggesting that the Nrf2/HO-1 axis may be necessary for the protective effects of irisin. Nrf2 is a crucial regulator of lipid peroxidation and ferroptosis [40]. Nrf2 is generally inactive because it is linked to the Kelch-like-ECH-associated protein 1 (Keap1). Nrf2 is released from Keap1 under periods of stress and migrates to the nucleus, triggering antioxidant response element- (ARE-) dependent genes. Most Nrf2 target genes are able to inhibit lipid peroxidation and ferroptosis [40]. HO-1 is a detoxifying enzyme in phase II that can be upregulated by Nrf2. The Nrf2/HO-1 axis appears to be implicated in mitochondrial oxidative stress and ferroptosis [41]. It has been shown that fingolimod alleviates Vitk3-induced cytotoxicity by reducing mitochondrial ROS generation via the Nrf2/HO-1 axis [42]. Another study proved that the Nrf2/HO-1 axis could simultaneously enhance CO generation and promote the expression of mitochondrial biosynthesis and antioxidant genes, leading to tolerance to doxorubicin-mediated mitochondrial damage and cardiomyocyte apoptosis [43]. These findings imply that Nrf2/HO-1 axis can modify mitochondrial structure and function to protect them from oxidative damage. Li et al. discovered that ferroptosis contributes to radiation-induced lung fibrosis (RILF) and that the ferroptosis blocker liproxstatin-1 ameliorates RILF by inhibiting TGF-1 via activation of the Nrf2/HO-1 pathway [44]. This study validated previous results that I/R or H/R produced ferroptosis in MLE-12 cells by blocking the Nrf2/HO-1 axis and boosting intracellular iron and lipid oxide accumulation in LIRI. However, irisin protects against ferroptosis and oxidative stress upon LIRI by elevating nuclear Nrf-2 and cytosolic HO-1 expression in vivo and in vitro. In addition, siRNA-mediated Nrf-2 knockdown reversed the beneficial effects of irisin on LIRI and HO-1 expression. These findings show that irisin inhibits lung I/R-induced ferroptosis and oxidative injury through the Nrf2/HO-1 axis.

Despite these findings, our research has several limitations. First, lung I/R injury can affect lung epithelial cells, lung bronchus epithelial cells, and endothelial cells. However, our in vitro experiments were limited to lung epithelial cells. Second, we focus on the effect of irisin on ferroptosis. Although we demonstrated the solid antiferroptosis actions of irisin by using the specific ferroptosis modulators such as Fer-1 and Fe-citrate, we were unable to test the role of irisin in other forms of cell death such as autophagy and necroptosis. Third, the precise mechanism by which irisin regulates the Nrf2/HO-1 signal axis needs further investigation.

## 5. Conclusions

**Figure 8:**
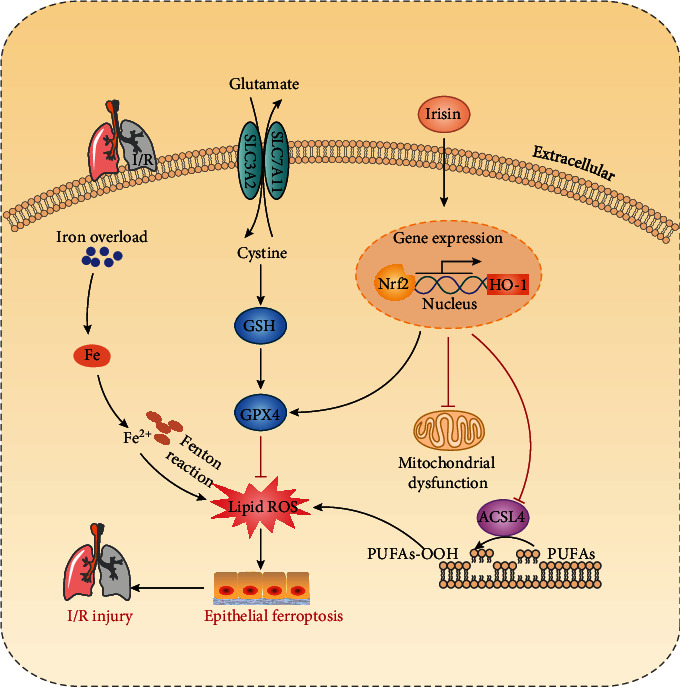
This graphic is intended to illustrate the role of the Nrf2/HO-1 signaling pathway in irisin's protection against lung I/R-induced ferroptosis. Lung I/R results in an accumulation of iron and lipid peroxidation, mitochondrial dysfunction, and a decrease in antioxidation systems such as GPX4 and glutathione, implying a role for ferroptosis in the pathogenesis of LIRI. Irisin substantially decreased ferroptosis, relieved LIRI, and enhanced mitochondrial function by activating the Nrf2/HO-1 signaling axis. Ferrostatin-1 (Fer-1), a ferroptosis inhibitor, and Fe-citrate (Fe), a ferroptosis inducer, may attenuate or accelerate ferroptosis in lung I/R injury, respectively.

In summary, as shown in Figure 8, our findings demonstrated that lung I/R induced ferroptosis via increased iron and lipid peroxidation, along with GSH depletion. Irisin effectively suppressed ferroptosis, alleviated LIRI, and improved mitochondria function via induction of the Nrf2/HO-1 signal axis. This study provides favorable evidence for the application of irisin in the treatment of LIRI.

## Data Availability

The [DATA TYPE] data used to support the findings of this study are included within the article and the supplementary information file(s).

## Supplementary Materials

Supplementary Materials
Figure 1: the effects of various irisin dose levels on LIRI. Mice were subjected to a sham operation or lung I/R (60 minutes of ischemia followed by 120 minutes of reperfusion) with or without irisin (Ir). (A) Representative images (200x) of HE staining in lung sections. (B) the degree of lung injury. The plasma PaO_2_ (C) and PaCO_2_ (D) were from mice subjected to different groups. ^∗^*P* < 0.05 vs. sham group, ^#^*P* < 0.05 vs. I/R group, and ^&^*P* < 0.05 vs. I/R+Ir 125 group. The data are presented as mean ± SD (*n* = 6).  Figure 2: effects of irisin on inflammatory factors in LIRI. Mice were subjected to a sham operation or lung I/R (60 minutes of ischemia followed by 120 minutes of reperfusion) with or without irisin (Ir), ferrostatin-1 (Fer-1), or Fe-citrate (III) (Fe) administration. The levels of TNF-*α* (A), IL-1*β* (B), and IL-6 (C) in BALF were analyzed using an ELISA assay. ^∗^*P* < 0.05 vs. sham group, ^#^*P* < 0.05 vs. I/R group, and ^&^*P* < 0.05 vs. Fe group. The data are presented as mean ± SD.  Figure 3: irisin inhibited H/R-induced cell damage dose dependently. MLE-12 cells were incubated with or without irisin at the beginning of reoxygenation. (A) Cell viability was determined using the CCK-8 assay kit. The levels of LDH (B) and MDA (C) in MLE-12 cells were analyzed using the corresponding kit. ^∗^*P* < 0.05 vs. Con group, ^#^*P* < 0.05 vs. H/R group, and ^&^*P* < 0.05 vs. Fe group. The data are presented as mean ± SD (*n* = 6).
