# Supplementary material for: Postconditioning with Irisin Attenuates Lung Ischemia/Reperfusion Injury by Suppressing Ferroptosis via Induction of the Nrf2/HO-1 Signal Axis
Source: Oxid Med Cell Longev. 2022 Mar 2;2022:9911167. doi: 10.1155/2022/9911167 (PMC8906956; doi:10.1155/2022/9911167)
Supplement: Supplementary Materials — Figure 1: the effects of various irisin dose levels on LIRI. Mice were subjected to a sham operation or lung I/R (60 minutes of ischemia followed by 120 minutes of reperfusion) with or without irisin (Ir). (A) Representative images (200x) of HE staining in lung sections. (B) the degree of lung injury. The plasma PaO2 (C) and PaCO2 (D) were from mice subjected to different groups. ∗P < 0.05 vs. sham group, #P < 0.05 vs. I/R group, and &P < 0.05 vs. I/R+Ir 125 group. The data are presented as mean ± SD (n = 6). Figure 2: effects of irisin on inflammatory factors in LIRI. Mice were subjected to a sham operation or lung I/R (60 minutes of ischemia followed by 120 minutes of reperfusion) with or without irisin (Ir), ferrostatin-1 (Fer-1), or Fe-citrate (III) (Fe) administration. The levels of TNF-α (A), IL-1β (B), and IL-6 (C) in BALF were analyzed using an ELISA assay. ∗P < 0.05 vs. sham group, #P < 0.05 vs. I/R group, and &P < 0.05 vs. Fe group. The data are presented as mean ± SD. Figure 3: irisin inhibited H/R-induced cell damage dose dependently. MLE-12 cells were incubated with or without irisin at the beginning of reoxygenation. (A) Cell viability was determined using the CCK-8 assay kit. The levels of LDH (B) and MDA (C) in MLE-12 cells were analyzed using the corresponding kit. ∗P < 0.05 vs. Con group, #P < 0.05 vs. H/R group, and &P < 0.05 vs. Fe group. The data are presented as mean ± SD (n = 6). [file 9911167.f1.docx]

**Supplementary materials**

1. **Materials and Methods**
   1. Blood gas measurement. After reperfusion, 0.3 ml of arterial blood was taken for blood gas measurement to measure arterial oxygen (PaO2) and carbon dioxide (PaCO_2_) concentrations using an ABL 800 analyzer (Radiometer, Denmark).
   2. Measurement of pro-inflammatory cytokines in BALF. The levels of tumor necrosis factor (TNF)-α, IL-1β, and IL-6 in BALF were determined by the enzyme-linked immunosorbent assay (ELISA) Kit (Beyotime, Shanghai, China) according to the manufacturer's protocols.

1.3 Assay for lactate dehydrogenase (LDH) release. The release of LDH into the medium was determined using the LDH release Assay Kit (Beyotime) according to the manufacturer's protocols. The absorbance at 450 nm was measured by a microplate reader (PerkinElmer, USA).

**Supplementary figures and figure legends**


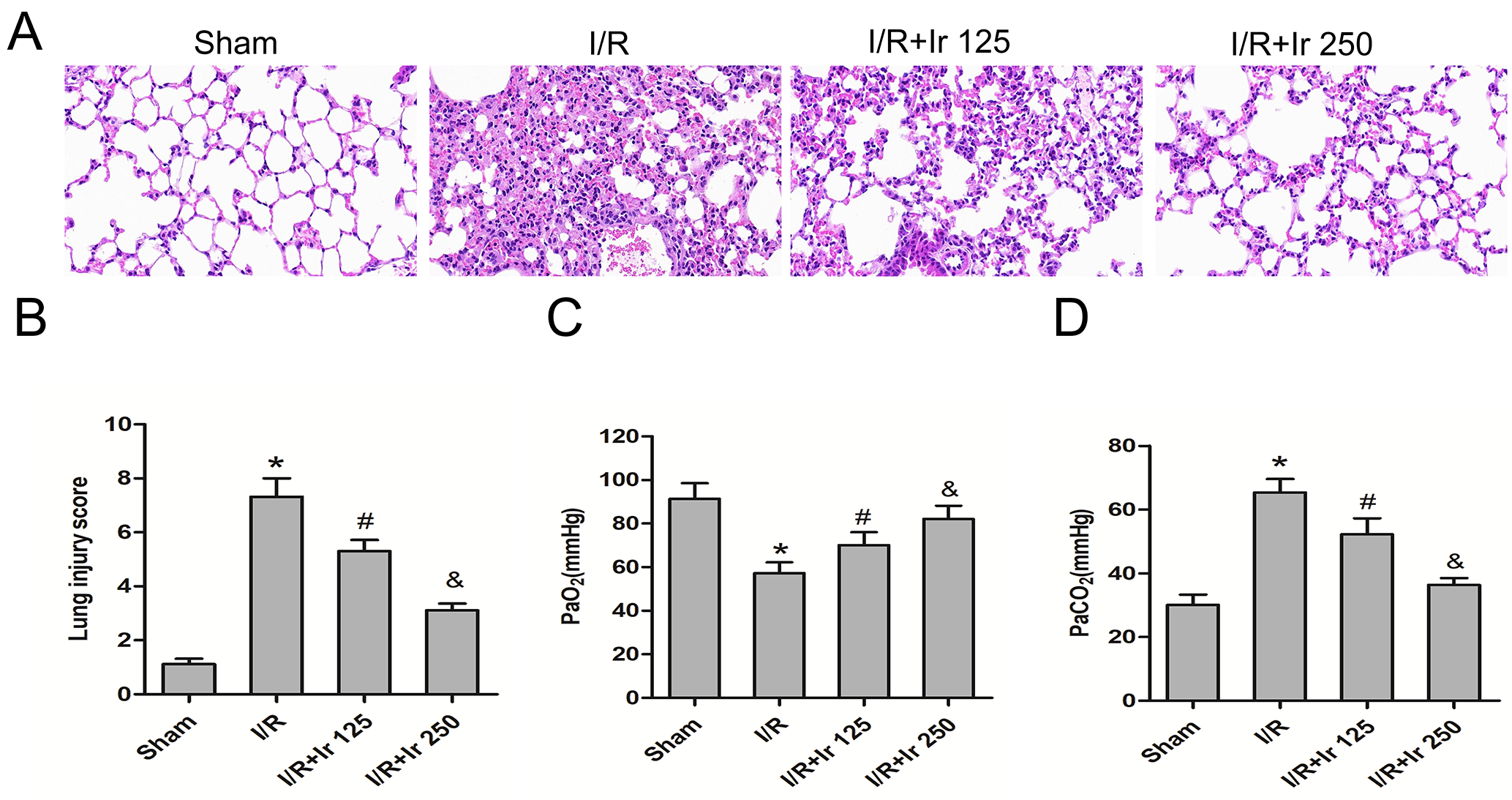


Figure 1. The effects of various irisin dose levels on LIRI. Mice were subjected to a sham operation or lung I/R (60 minutes of ischemia followed by 120 minutes of reperfusion) with or without irisin (Ir). (A) Representative images (200×) of HE staining in lung sections. (B) the degree of lung injury. The plasma PaO_2_ (C) and PaCO_2_ (D) were from mice subjected to different groups. . *P < 0.05 vs. Sham group, ^#^P < 0.05 vs. I/R group, and ^&^P < 0.05 vs. I/R+Ir 125 group. The data are presented as mean ± SD (n = 6).


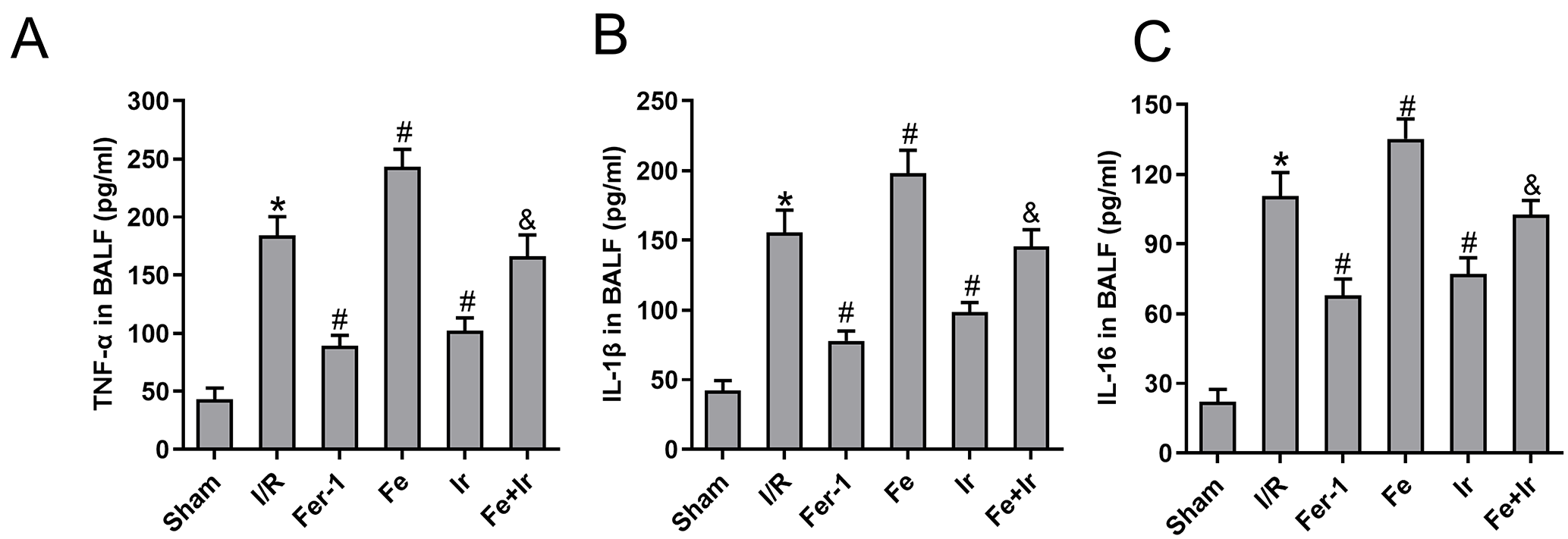


Figure 2. Effects of irisin on inflammatory factors in LIRI. Mice were were subjected to a sham operation or lung I/R (60 minutes of ischemia followed by 120 minutes of reperfusion) with or without irisin (Ir), ferrostatin-1 (Fer-1) or Fe-citrate (III) (Fe) administration. The levels of TNF-α (A), IL-1β (B) and IL-6 (C) in BALF were analyzed using an ELISA assay. *P < 0.05 vs. Sham group, ^#^P < 0.05 vs. I/R group, and ^&^P < 0.05 vs. Fe group. The data are presented as mean ± SD.


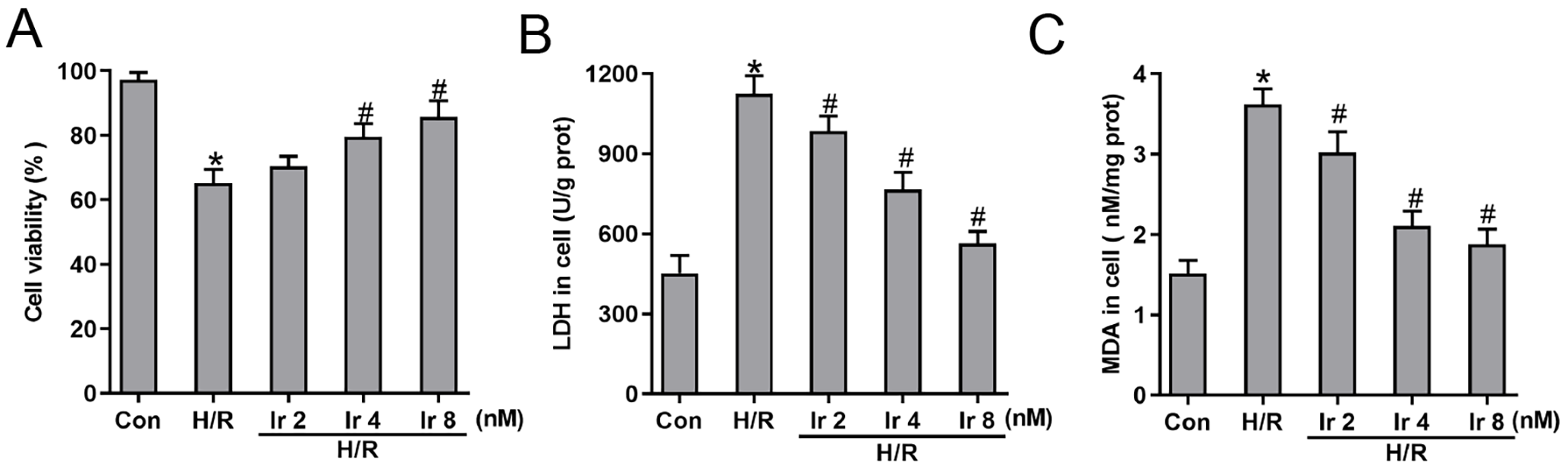


Figure 3. Irisin inhibited H/R-induced cell damage dose-dependently. MLE-12 cells were incubated with or without irisin at the beginning of reoxygenation. (A) Cell viability was determined using the CCK-8 assay kit. The levels of LDH (B) and MDA (C) in MLE-12 cells were analyzed using the corresponding kit. *P < 0.05 vs. Con group, ^#^P < 0.05 vs. I/R group, and ^&^P < 0.05 vs. Fe group. The data are presented as mean ± SD (n = 6).
